# Awareness of Vaccination against Respiratory Tract Diseases, Including Pneumonia, Influenza, and COVID-19 in Patients with Diabetes Mellitus

**DOI:** 10.1155/2022/1389137

**Published:** 2022-08-10

**Authors:** Baris Karagun, Mehtap Evran, Fulya Odabas, Gamze Akkus, Behice Kurtaran, Murat Sert, Tamer Tetiker

**Affiliations:** ^1^Cukurova University, Faculty of Medicine, Division of Endocrinology, Adana, Turkey; ^2^Cukurova University, Faculty of Medicine, Division of Infectious Diseases, Adana, Turkey

## Abstract

Patients with diabetes have an increased risk of severe acute respiratory infections, and vaccination is their life-saving option. This study aimed to investigate the interest and knowledge of patients about influenza, pneumonia, and COVID-19(coronavirus disease 2019) vaccines. *Materials*. We handed out a questionnaire to patients with diabetes who were admitted to the endocrinology clinic between April and August 2021. The questionnaire collected information on demographic data, knowledge about respiratory tract disease vaccines, and hesitancy about vaccines. *Results*. Four hundred twenty-four patients (female = 256, male = 168) enrolled in the study. In this study, 148 (34.9%) participants were vaccinated against pneumonia, 155 (36.6%) against flu, and 312 (73.6%) against COVID-19. In addition, antivaccination sentiment was recorded in 8.7% of patients with diabetes. We found that participants in the study primarily rely on doctors as the source of information about vaccines (doctor (46.7%), nurse (1.2%), television (8.7%), friend/neighbour (8.7%), and others (2.6%)). The rate of vaccination was statistically higher than the presence of comorbid diseases. *Conclusions*. We examined the vaccine awareness of patients with diabetes and investigated factors affecting it. İt was determined that vaccination awareness is affected by many factors, especially comorbid diseases and educational status. The study showed that patients primarily relied on doctors as their source of information for vaccination. Doctor-centered vaccination promotion programmes can increase the rate of vaccination.

## 1. Introduction

Diabetes mellitus is a serious health problem that leads to high medical care costs, low quality of life, and increased mortality. The prevalence of diabetes in societies is rising with lifestyle changes. The International Diabetes Federation recorded in 2017 around 451 million (18–99 years old) people with diabetes worldwide and estimated that this number will reach 693 million in 2045 [1]. Vaccination is a life-saving tool in preventing infectious diseases [2]. Vaccines not only provide individual protection but can also alleviate the economic burden of infectious diseases [3]. The pathogenesis of infectious diseases, which are more common in patients with diabetes, includes hyperglycaemia-related increased virulence, decreased interleukin response and chemotaxis and impaired phagocytic activity, and leukocyte function [4]. A study involving 100,000 diabetic patients showed that patients with type 1 and type 2 diabetes have higher risks of infectious diseases and risks of hospitalisation and death due to these diseases compared with the control group without diabetes. In addition, pneumonia was the leading cause of hospitalisation in elderly patients among infectious diseases [5]. Because influenza and pneumococcal immunisations have been proven effective on morbidity and mortality related to these diseases in patients with diabetes, all patients with diabetes, especially those who develop cardiac and renal complications, should be vaccinated [6]. The SARS-CoV-2 pandemic and its associated COVID-19 disease have higher morbidity and mortality rates, especially in elderly patients and patients with chronic conditions such as diabetes [7].

This study aimed to determine vaccine awareness of patients with diabetes. Because of mortality rates due to respiratory tract infections, we wanted to emphasise the rate of vaccine adoption of patients with diabetes and the factors affecting this situation.

## 2. Methods

We included 424 patients (female = 256, male = 168) with diabetes who were admitted to the Endocrinology Department of Çukurova University Hospital between April and August 2021 in this cross-sectional study. We provided a questionnaire form to the patients using the face-to-face interview technique. We obtained verbal and written consent from all subjects. The questionnaire form included 21 questions that evaluate the sociodemographic characteristics of patients and behavioural information about adult immunisation. We collected data on age, gender, type of diabetes, glycated haemoglobins (HbA1c), duration of diabetes, and current treatment method of the patient. We excluded patients who did not want to participate in the study, who were under the age of 18, and who were pregnant.

In this study, we referred those who have not been vaccinated or who have missed their vaccination to the vaccine polyclinic.

### 2.1. Statistical Analysis

We expressed categorical variables as numbers and percentages, whereas continuous variables were expressed as the mean and standard deviation or as the median and minimum-maximum where appropriate. We used the *χ*^2^ test to compare categorical variables between groups. We confirmed the normality of distribution for continuous variables using the Shapiro–Wilk test. For comparison of continuous variables between two responses (s16), we used Student's *t*-test or the Mann–Whitney U test depending on whether the statistical hypotheses were fulfilled or not. For comparison of more than two responses (s16 s17), we used one-way ANOVA or the Kruskal–Wallis test depending on whether the statistical hypotheses were fulfilled or not. For normally distributed data, regarding the homogeneity of variances, we used the Tukey and Games–Howell tests for multiple comparisons of groups. For non-normally distributed data, we used the Bonferroni-adjusted Mann–Whitney U test for multiple comparisons of groups. We performed all analyses using the IBM SPSS Statistics Version 20.0 statistical software package (IBM SPSS Statistics for Windows version 20.0, Armonk, NY, IBM Corp.). The statistical level of significance for all tests was set at .05.

## 3. Results

A total of 424 (168 males (39.6%) and 256 females (60.4%)) patients participated in our study. The mean age was 53.6 ± 12.86 years (range, 18–83); disease duration was 10.9 ± 8.6 years (range, 1–45); the HbA1c (%) level of the patients was 8.16 ± 1.96 (range, 5.5–16). In terms of educational status, 192 (45.3%) reached elementary school, 64 (15.1%) middle school, 79 (18.6%) high school, and 60 (14.2%) university level. Twenty-nine (6.8%) patients were illiterate. Thirty-nine (9.2%) patients were diagnosed with type 1 diabetes and 385 (90.8%) with type 2 diabetes (Table 1).

In the study, 148 (34.9%) were vaccinated against pneumonia, 155 (36.6%) against flu, and 312 (73.6%) against COVID-19 (Table 2).

We found no significant correlation between patients' gender and prior vaccination against pneumonia (*p*=0.368), COVID-19 (*p*=0.423), and influenza (*p*=0.566). In addition, we found no significant correlation between the patients' education level and prior vaccination against pneumonia (*p*=0.915) and COVID-19 (*p*=0.160); however, we found a significant correlation between education and prior vaccination against influenza (*p*=0.017).

We found no significant correlation between education level, gender, and the answer to the question ‘Do you get the recommended vaccinations? (*p*=0.102, *p*=0.602).' In addition, we found no significant correlation between the diabetes type and the answer to the question ‘Do you get the recommended vaccinations? (*p*=0.403) (Table 3).'

Considering the vaccination rate according to the presence of additional diseases other than diabetes, we found that pneumonia, COVID-19, and influenza vaccination rates were statistically significantly higher (*p*=0.017, *p*=0.08, and *p*=0.02, respectively). Among these additional diseases, hypertension ranked first with 42.2%, followed by cardiovascular diseases with 14.6%.

In our study, antivaccination sentiment was found among 8.7% of patients with diabetes. In addition, we found that the primary reason for those who were against the vaccine was the belief that vaccines are harmful.

## 4. Discussion

Patients with diabetes may have increased morbidity and mortality rates from infection because of abnormalities in immune function. Furthermore, influenza and pneumococcal diseases lead to higher mortality rates in patients with diabetes [6]. Vaccination is one of the most effective and safest methods of preventing infectious diseases. Its efficacy against influenza, pneumonia, and COVID-19 has been proven to reduce mortality and morbidity rates [8–10]. In our study, vaccination rates against influenza and pneumococcal diseases were 36.6% and 34.9%, respectively, which were higher compared with those from other studies on vaccine awareness in diabetes conducted in Turkey [11–13] but still lower than the global targets [14]. According to the Centers for Disease Control and Prevention, within the scope of ‘Healthy People 2020', the targeted flu vaccination rate is 70% for adults over the age of 18 years and 90% for healthcare professionals [14].

Vaccine awareness plays an important role in overcoming barriers against vaccines, and most sociodemographic and physical variables may be significantly related to influenza vaccine hesitancy [15]. One hundred eighty-five (43.6%) participants in our study had previous knowledge about pneumonia vaccines and 274 (64.6%) about influenza vaccines. Patients learned about vaccines from doctors (198 (46.7%)) first, followed by television (37 (8.7%)), friends/neighbours (8.7%), and nurses (1.2%). The epidemiological study ‘diaVAX' showed that patients with diabetes with severe illness are less likely to be vaccinated and that increased awareness of physicians may help improve vaccination rates against influenza and pneumococcal diseases [16]. In our study, patients primarily relied on doctors in obtaining information about the vaccine, and as demonstrated in the ‘diaVAX' study, physician education about the vaccine proved to be advantageous to society.

COVID-19 appeared, firstly, in Wuhan, Hubei Province, China, at the end of 2019, and since then, spread globally and has been reported rapidly in various countries [17]. The COVID-19 pandemic has had devastating effects on every aspect of global health and worsened quality of life where it was reported [18]. In a multicentric study, it was shown that the pandemic changed the psychological well-being of populations in different manners [19]. The COVID-19 pandemic had a higher rate of negative health consequences in those with chronic diseases [7]. In patients with diabetes who have a serious chronic condition, interest in vaccination is life-saving to protect them from the devastating effects of the pandemic [10]. In our study, we also investigate the awareness of patients with diabetes about the COVID-19 vaccine, based on the effect of COVID 19 on psychological well-being. COVID-19 is associated with increased mortality rates and severity of disease in patients with diabetes [20, 21]. In our study, 84 (19.8%) participants were previously infected with SARS-CoV-2. Three hundred twelve (73.6%) were vaccinated against COVID-19. On the basis of these results, we can assume that the COVID-19 vaccine acceptance in our study was higher than that shown in other studies from other countries [22, 23]. This can be attributed to the fact that doctors were given an important role in the COVID-19 vaccine incentive programme in our country and that the recommendations received from doctors are more convincing, as shown in our study. Sapkota et al. showed that the COVID-19 epidemic demonstrates chaotic behaviour, and it is difficult to predict the dynamics of the pandemic in the long term [24]. Based on these data, health policies should be constructed based on the effects of the physician awareness effect on population behaviour. Existing diseases affect the interest in vaccines. Hao et al. showed that psychiatric patients have a high acceptance and willingness to pay for the COVID-19 vaccine [25]. In one study from Vietnam, it was shown that some conditions were associated with a higher likelihood of acceptance and willingness for the vaccine. These conditions were associated with a higher income, having children, self-perceived risk of COVID-19 infection, and perceived risk to friends. Payment for COVID-19 vaccines may cause another barrier to adequate coverage, which was highlighted in this study [26]. A possible explanation for the high vaccination rates in our study may be strong national strategies to promote vaccination and make vaccination free and easily accessible in our country. These national strategies included providing patients in high-risk groups with appropriate and comprehensive information from healthcare professionals. Free vaccination policy through the national immunisation campaign in Turkey has undoubtedly contributed to the increase in vaccination. Pneumococcal and influenza vaccines are provided free of charge in primary care to those with chronic diseases such as diabetes. In our clinic, informing about vaccines and directing diabetic patients to the vaccination outpatient clinic for pneumococcal and influenza vaccination also contributed to the high vaccination rates in our study.

Today, we know that education level has a positive effect on vaccination [27, 28]. In terms of correlations between education level and vaccination in our study, we found a statistically significant correlation between influenza and education level, implying that as the education level increases, the demand for the vaccine increases. The same was true for the COVID-19 vaccine, which had a positive, although not statistically significant correlation, with education level. This can be attributed to the increasing level of education and the fact that individuals research and learn about the scientific benefits of vaccination.

In terms of vaccination rates according to the types of diabetes, we found that more patients with type 1 diabetes were vaccinated. Although this was not statistically significant, this was one of the remarkable findings in our study. Another remarkable finding that can be drawn from this study is that the presence of comorbid disease increases the preference for vaccination. Considering that these comorbid diseases increase mortality rates in patients with diabetes [29], this result was promising.

## 5. Conclusion

In our study, we examined the factors affecting vaccine awareness of patients with diabetes and investigated their effects. One of the most remarkable findings was that patients primarily relied on doctors as a source of information. This result underlines the importance of raising physician awareness as a first step in vaccination. Thus, we believe that doctor-centered vaccine incentive programmes can achieve more success in preventing respiratory tract infections, which can lead to mortality in patients with diabetes, to some extent.

### 5.1. Study Limitations

In our country, the COVID-19 vaccine was gradually provided to citizens by age groups, starting from the advanced age group, because of the lack of supply at the onset of the pandemic. For this reason, young patients with diabetes who were not yet vaccinated during the survey were flagged as unvaccinated for COVID-19 during the study. The study was conducted in a tertiary health facility in Adana, and it may not reflect the country's actual vaccination rate.

## Figures and Tables

**Table 1:** Summary of the demographic and biochemical data of the patients.

Patients (*N* = 424)		
Sex: F/M		256/168
Age (mean, years)		53.6 ± 12.86
Duration of diabetes mellitus (mean, years)		10.9 ± 8.6
Serum level of HbA1c (mean, mg/dl)		8.16 ± 1.96

Educational status, *N* (%)		Illiterate, 29 (6.8%)
		Elementary school, 192 (45.3%)
		Middle school, 64 (15.1%)
		High school, 79 (18.6%)
		University, 60 (14.2%)

Type of diabetes, *N* (%)	Type 1	39 (9.2%)
	Type 2	385 (90.8%)

Type of the treatment, *N* (%)	Diet	26 (6.1%)
	OAD	191 (45%)
	Insulin	94 (22.2%)
	OAD + insulin	113 (26.7%)

**Table 2:** Patient's vaccination and disease history.

Have you ever been vaccinated for pneumonia before? *N* (%)	Yes	148 (34.9%)
	No	253 (59.7%)
	I do not remember	23 (5.4%)
Have you ever been vaccinated for influenza before? *N* (%)	Yes	155 (36.6%)
	No	261 (61.6%)
	I do not remember	8 (1.9%)

Have you ever been vaccinated for COVID-19 before? *N* (%)	Yes	312 (73.6%)
	No	112 (26.4%)

Have you ever had COVID-19 infection before?	Yes	84 (19.8%)
	No	340 (80.2%)

Have you ever had pneumonia?	Yes	44 (10.4%)
	No	380 (89.6%)

**Table 3:** Vaccine awareness of patients with diabetes.

Do you have information about the pneumonia vaccine? *N* (%)	Yes	185 (43.6%)
	No	239 (56.4%)
Do you have information about the influenza vaccine? *N* (%)	Yes	274 (64.6%)
	No	150 (35.4%)

Whom did you get information about the vaccine? *N* (%)	Doctor	198 (46.7%)
	Nurse	5 (1.2%)
	Television	37 (8.7%)
	Friend/neighbour	37 (8.7%)
	Others	11 (2.6%)

Did you know that the flu vaccine is repeated annually?	Yes	266 (62.7%)
	No	158 (37.3%)

Did you know that the pneumonia vaccine is a single dose?	Yes	154 (36.3%)
	No	270 (63.7%)

Should people with diabetes get vaccinated?	Yes	335 (79%)
	No	24 (5.7%)
	I do not know	65 (15.3%)

Do you get the recommended vaccinations?	Yes	387 (91.3%)
	No	37 (8.7%)

If your answer to the last question is no, what is the reason?	I do not think the vaccines are effective	10 (2.4%)
	I think vaccines are as harmful as good	12 (2.8%)
	I am impressed by the antivaccine views in the media	4 (0.9%)
	I do not think it is necessary to get vaccinated	9 (2.1%)
	I am afraid of allergic reactions after vaccination	2 (0.5%)

## Data Availability

The data used to support the findings of this study are included within the article.
